# Supplementary material for: Revealing the status of Orbicella: Main reef-builder of Morrocoy National Park and Cuare Wildlife Refuge, Venezuela, Southern Caribbean
Source: PLoS One. 2025 Feb 7;20(2):e0317728. doi: 10.1371/journal.pone.0317728 (PMC11805429; doi:10.1371/journal.pone.0317728)
Supplement: S1 Table — PERMANOVA under a mixed-effect linear model with two fixed factors (sampling period and sector) and a random factor (reef) nested within sector based on Euclidean distance. (DOCX) [file pone.0317728.s001.docx]

**Revealing the status of *Orbicella*: Main reef-builder of Morrocoy National Park and Cuare Wildlife Refuge, Venezuela, Southern Caribbean**

Anaurora Yranzo**-**Duque, Ana Teresa Herrera-Reveles, Estrella Villamizar, Francoise Cabada-Blanco, Jeannette Pérez-Benítez, Hazael Boadas, José G. Rodríguez-Quintal, Carlos Pereira, Samuel Narciso, Freddy A. Bustillos

Supplementary Table 1. *Orbicella annularis* and *Orbicella faveolata* live cover in Morrocoy National Park and Cuare Wild life Refuge, Venezuela (period 2018-2020). PERMANOVA under a mixed-effect linear model with two fixed factors (sampling period and sector) and a random factor (reef) nested within sector based on Euclidean distance (gl: degrees of freedom. SC: sum of squares. MC: mean of the squares. F: statistic value. p: probability estimated by permutations. % CV: percentage of the variation component attributable to each source). Sampling period - four levels: July 2018, November 2018, July 2019 and January 2020; Sector- five levels: Northern, Center, Southern, Banks and Refuge. Reef: 12 levels. N= 140 transects.

| Linear cover | Source | gl | SC | SM | F | p | %CV |
| --- | --- | --- | --- | --- | --- | --- | --- |
| ***O. ann*** | Period | 3 | 0.34212 | 0.11404 | 0.9581 | 0.4304 | 0 |
|  | Sector | 4 | 3.5146 | 0.87865 | 9.8804 | 0.0333 | 24.45 |
|  | Reef (sector) | 7 | 0.62595 | 8.94E-02 | 1.2474 | 0.2813 | 1.28 |
|  | Period x Sector | 11 | 0.72373 | 6.58E-02 | 0.55227 | 0.848 | 0 |
|  | Period x Reef(sector) | 20 | 2.3832 | 0.11916 | 1.6622 | 0.0536 | 13.17 |
|  | Residuals | 96 | 6.8818 | 7.17E-02 |  |  | 61.11 |
|  | *Total* | *141* | *14.574* |  |  |  | *100.00* |
| ***O. fav*** | Period | 3 | 0.53108 | 0.17703 | 0.88291 | 0.4625 | 0 |
|  | Sector | 4 | 10.755 | 2.6888 | 10.277 | 0.0036 | 35.73 |
|  | Reef(sector) | 7 | 1.8598 | 0.26569 | 2.2141 | 0.0416 | 4.99 |
|  | Period x Sector | 11 | 0.83001 | 7.55E-02 | 0.37599 | 0.948 | 0 |
|  | Period x Reef(sector) | 20 | 4.0146 | 0.20073 | 1.6728 | 0.0528 | 10.64 |
|  | Residuals | 96 | 11.52 | 0.12 |  |  | 48.63 |
|  | *Total* | *141* | *30.573* |  |  |  | *100.00* |
