# Supplementary material for: Revealing the status of Orbicella: Main reef-builder of Morrocoy National Park and Cuare Wildlife Refuge, Venezuela, Southern Caribbean
Source: PLoS One. 2025 Feb 7;20(2):e0317728. doi: 10.1371/journal.pone.0317728 (PMC11805429; doi:10.1371/journal.pone.0317728)
Supplement: S2 Table — (DOCX) [file pone.0317728.s002.docx]

Revealing the status of *Orbicella*: Main reef-builder of Morrocoy National Park and Cuare Wildlife Refuge, Venezuela, Southern Caribbean

Anaurora Yranzo**-**Duque, Ana Teresa Herrera-Reveles, Estrella Villamizar, Francoise Cabada-Blanco, Jeannette Pérez-Benítez, Hazael Boadas, José G. Rodríguez-Quintal, Carlos Pereira, Samuel Narciso, Freddy A. Bustillos

Supplementary Table 2**.** Synthesis of previous live cover reports for *Orbicella annularis* *(O.ann) and O. faveolata (O.fav)* in Morrocoy National Park (northern, center and southern sectors) and Cuare Wildlife Refuge (Refuge sector) Venezuela. Survey year in parenthesis. ND: No data. B: before the massive mortality; A: after the massive mortality.

| LIVE COVER (%) BY SECTOR-REEF | | | | | | | | | | | | | | | | | | | | |
| --- | --- | --- | --- | --- | --- | --- | --- | --- | --- | --- | --- | --- | --- | --- | --- | --- | --- | --- | --- | --- |
| Species | Northern | | Center | | | | | Southern | | | | | | Refuge | | | | | | References |
|  | Peraza | | Sombrero | | | Caiman | | Playuelita | | | Mero | | | Norte | | | Sur | | |  |
| ***O.ann*** | ND | | B  15.55 to  22.02  (1996-2000) | | A  25% (1997)  20% (2005) | B  43.35 (1994)  37.5 (1995) | A  ˂5 (1996,2000)  6.84 (2004) | B  ND | | A  12.3 (2000) | B  1.07 to 14.81  (1996) | | A  ˂3 (1999) | B  ND | | A  4.14 to  14.54 (2003,2004) | B  ND | | A  2.71 (2003,  2004) | 1,2,3,24,31 |
|  | **0** | | **4.44** | | | **0.69** | | **0** | | | **1.92** | | | **5.68** | | | **4.62** | | | **Current study**  **(2018-2020)** |
| ***O.fav*** | ND | 3.9  (2009) | ND | 9.2%  (2005) | | ND | 14.7 (2004) | ND | ND | | ND | ˂ 5 (2005) | | ND | 11.62 and 73.62% of relative cover  (2003,  2004) | | ND | 14.17 (2003,  2004) | | 2,3,4,24 |
|  | **13.02** | | **23.59** | | | **13.20** | | **6.04** | | | **4.19** | | | **43.32 and** **66.09% of relative cover** | | | **31** | | | **Current study**  **(2018-2020)** |

**O.annularis* complex cover in Caiman= 32%(1993) and 28% (1996) prior to the mortality event) and ˂ 1 (2003) after the event (5).

References

1. Villamizar E. Estructura de una comunidad arrecifal en Falcón, Venezuela antes y después de una mortalidad masiva. Revista de Biología Tropical. 2000; 47:19-3.
2. Cróquer A, Debrot D, Klein E, Kurten M, Rodríguez S, Bastidas C. What can two years of monitoring tell us about Venezuelan coral reefs? The Southern Tropical America node of the Global Coral Reef Monitoring Network (STA-GCRMN). Rev Biol Trop. 2010; 58, 51-65.
3. Del Mónaco C, Villamizar E, Narciso, S. Selectividad de presas de Coralliophila abbreviata y C. caribaea en arrecifes coralinos del Parque Nacional Morrocoy, Venezuela: una aproximación experimental. Latin American J Aquat Res. 2010; 38: 57-70.
4. López-Ordaz A, Rodríguez-Quintal J. Ictiofauna asociada a un arrecife somero en el Parque Nacional Morrocoy, Venezuela. Rev Biol Trop. 2010; 58:163-174.
5. Bastidas C, Croquer, A, Bone, D. Shift of dominant species after a mass mortality on a Caribbean reef. Proceedings of 10th *International Coral Reef Symposium*. 2006; 989-993.
