# Supplementary material for: Revealing the status of Orbicella: Main reef-builder of Morrocoy National Park and Cuare Wildlife Refuge, Venezuela, Southern Caribbean
Source: PLoS One. 2025 Feb 7;20(2):e0317728. doi: 10.1371/journal.pone.0317728 (PMC11805429; doi:10.1371/journal.pone.0317728)
Supplement: S3 Table — PERMANOVA under a mixed-effect linear model using two fixed factors (sampling period and sector) and a random factor (reef) nested within sector based on Euclidian distance. (DOCX) [file pone.0317728.s003.docx]

Revealing the status of *Orbicella*: Main reef-builder of Morrocoy National Park and Cuare Wildlife Refuge, Venezuela, Southern Caribbean

Anaurora Yranzo**-**Duque, Ana Teresa Herrera-Reveles, Estrella Villamizar, Francoise Cabada-Blanco, Jeannette Pérez-Benítez, Hazael Boadas, José G. Rodríguez-Quintal, Carlos Pereira, Samuel Narciso, Freddy A. Bustillos

Supplementary Table 3. Tissue mortality of *O. faveolata* colonies in Morrocoy National Park and Cuare Wildlife Refuge, Venezuela (2018-2020). PERMANOVA under a mixed-effect linear model using two fixed factors (sampling period and sector) and a random factor (reef) nested within sector based on Euclidian distance (gl: degrees freedom. SC: sum of squares. MC: mean of squares. F: statistic value. p: probability estimated by permutations. % CV: percentage of the variation component attributable to each source). Sampling period - four levels: July 2018, November 2018, July 2019 and January 2020; Sector- five levels: Northern, Center, Southern, Banks and Refuge; Reef: 12 levels. N= 498 colonies

| %Mortality | Source | gl | SC | SM | F | p | %CV |
| --- | --- | --- | --- | --- | --- | --- | --- |
| **Recent** | Period | 3 | 947.78 | 315.93 | 4.2442 | 0.0455 | 4.98 |
|  | Sector | 4 | 2575.4 | 643.86 | 5.9679 | 0.0208 | 9.06 |
|  | Reef(sector) | 8 | 915.94 | 1.14E+02 | 1.7211 | 0.1051 | 1.79 |
|  | Period x Sector | 10 | 1327.9 | 1.33E+02 | 1.708 | 0.1827 | 2.95 |
|  | Period x Reef(sector) | 15 | 1210 | 80.668 | 1.2126 | 0.2565 | 1.36 |
|  | Residuals | 458 | 30467 | 6.65E+01 |  |  | 79.86 |
|  | *Total* | *498* | *41233* |  |  |  |  |
| **Old** | Period | 3 | 10520 | 3506.7 | 1.6382 | 0.2362 | 1.84 |
|  | Sector | 4 | 9410.7 | 2352.7 | 0.48163 | 0.7035 | 0.00 |
|  | Reef(sector) | 8 | 44125 | 5515.6 | 5.9014 | 0.0001 | 11.17 |
|  | Period x Sector | 10 | 12337 | 1.23E+03 | 0.46639 | 0.8671 | 0.00 |
|  | Period x Reef(sector) | 15 | 46352 | 3090.1 | 3.3063 | 0.0001 | 13.60 |
|  | Residuals | 458 | 428060 | 934.63 |  |  | 73.38 |
|  | *Total* | *498* | *567130* |  |  |  |  |
| *Total* | Period | 3 | 6772.3 | 2257.4 | 0.90842 | 0.4429 | 0.00 |
|  | Sector | 4 | 1848.1 | 462.01 | 0.10117 | 0.968 | 0.00 |
|  | Reef(sector) | 8 | 41180 | 5147.4 | 5.5342 | 0.0001 | 10.20 |
|  | Period x Sector | 10 | 9831.1 | 983.11 | 0.31354 | 0.955 | 0.00 |
|  | Period x Reef(sector) | 15 | 55639 | 3709.2 | 3.9879 | 0.0001 | 17.39 |
|  | Residuals | 458 | 425990 | 930.12 |  |  | 72.41 |
|  | *Total* | *498* | *558020* |  |  |  |  |
