# Supplementary material for: Revealing the status of Orbicella: Main reef-builder of Morrocoy National Park and Cuare Wildlife Refuge, Venezuela, Southern Caribbean
Source: PLoS One. 2025 Feb 7;20(2):e0317728. doi: 10.1371/journal.pone.0317728 (PMC11805429; doi:10.1371/journal.pone.0317728)
Supplement: S4 Table — PERMANOVA under a linear model with two fixed factors (sampling period and sector) and a random factor (reef) nested to the sector fixed factor. (DOCX) [file pone.0317728.s004.docx]

Revealing the status of *Orbicella*: Main reef-builder of Morrocoy National Park and Cuare Wildlife Refuge, Venezuela, Southern Caribbean

Anaurora Yranzo**-**Duque, Ana Teresa Herrera-Reveles, Estrella Villamizar, Francoise Cabada-Blanco, Jeannette Pérez-Benítez, Hazael Boadas, José G. Rodríguez-Quintal, Carlos Pereira, Samuel Narciso, Freddy A. Bustillos

Supplementary Table 4. Density of *Orbicella faveolata* colonies in reproductive and non-reproductive sizes in Morrocoy National Park and Cuare Wildlife Refuge, Venezuela (2018-2020). PERMANOVA under a linear model with two fixed factors (sampling period and sector) and a random factor (reef) nested to the sector fixed factor. (gl: degrees of freedom, SC: sum of squares, MC: mean of squares, F: value of the statistic, p: probability estimated by permutations, %CV: percentage of the variation component attributable to each source). N= 124 transects

|  | Source | gl | SC | SM | F | p | %CV |
| --- | --- | --- | --- | --- | --- | --- | --- |
| **Reproductive** | Period | 3 | 10,891 | 3,6304 | 2,081 | 0,1422 | 1,78 |
|  | Sector | 4 | 185,27 | 46,317 | 7,3295 | 0,0209 | 46,37 |
|  | Reef (sector) | 7 | 47,97 | 6,8528 | 5,0347 | 0,0004 | 14,00 |
|  | Period x Sector | 10 | 15,959 | 1,5959 | 0,91437 | 0,5468 | 0,00 |
|  | Period x Reef(sector) | 16 | 27,926 | 1,7454 | 1,2823 | 0,2309 | 3,21 |
|  | Residuals | 84 | 114,33 | 1,3611 |  |  | 34,64 |
|  | *Total* | *124* | *442,85* |  |  |  |  |
| **Non reproductive** | Period | 3 | 20,237 | 6,7457 | 1,3009 | 0,3039 | 1,25 |
|  | Sector | 4 | 62,85 | 15,712 | 2,0132 | 0,1723 | 7,79 |
|  | Reef (sector) | 7 | 58,313 | 8,3304 | 2,8523 | 0,0106 | 11,71 |
|  | Period x Sector | 10 | 41,304 | 4,1304 | 0,79584 | 0,629 | 0,00 |
|  | Period x Reef(sector) | 16 | 83,05 | 5,1906 | 1,7772 | 0,0507 | 16,12 |
|  | Residuals | 84 | 245,33 | 2,9206 |  |  | 63,14 |
|  | *Total* | *124* | *551,81* |  |  |  |  |
