# Supplementary material for: Revealing the status of Orbicella: Main reef-builder of Morrocoy National Park and Cuare Wildlife Refuge, Venezuela, Southern Caribbean
Source: PLoS One. 2025 Feb 7;20(2):e0317728. doi: 10.1371/journal.pone.0317728 (PMC11805429; doi:10.1371/journal.pone.0317728)
Supplement: S5 Table — Results for the simple linear regression in each period (1-July 2018, 2-November 2018, 3-July 2019 and 4-January 2020). (DOCX) [file pone.0317728.s005.docx]

**Revealing the status of *Orbicella*: Main reef-builder of Morrocoy National Park and Cuare Wildlife Refuge, Venezuela, Southern Caribbean**

Anaurora Yranzo**-**Duque, Ana Teresa Herrera-Reveles, Estrella Villamizar, Francoise Cabada-Blanco, Jeannette Pérez-Benítez, Hazael Boadas, José G. Rodríguez-Quintal, Carlos Pereira, Samuel Narciso, Freddy A. Bustillos

Supplementary Table 5. Total mortality (%) and sizes of *Orbicella faveolata* colonies in Morrocoy National Park and Cuare Wildlife Refuge, Venezuela (2018-2020). Results for the simple linear regression in each period (July 2018, n=147; November 2018, n=77); July 2019, n=136 and January 2020, n=120).

| Season | Coefficients | Estimate | Standar Error | t value | p-level | Regression Results |
| --- | --- | --- | --- | --- | --- | --- |
| **jul-18** | Intercept | -2.1407 | 0.4337 | -4.936 | 2.17E-06 | R^2^= 0.4807 F = 134.01 p = 0.000002 |
|  | Size | 1.0916 | 0.0943 | 11.576 | 2.00E-06 |  |
|  |  |  |  |  |  |  |
| **nov-18** | Intercept | -3.1363 | 0.7563 | -4.147 | 8.79E-05 | R^2^= 0.4510 F = 61.6 p = 2.313e-11 |
|  | Size | 1.4885 | 0.1896 | 7.849 | 2.31E-11 |  |
|  |  |  |  |  |  |  |
| **jul-19** | Intercept | -3.3514 | 0.43634 | -7.681 | 2.97E-12 | R^2^= 0.5963 F = 197.96 p = 0.000002 |
|  | Size | 1.3064 | 0.09285 | 14.07 | 2.00E-06 |  |
|  |  |  |  |  |  |  |
| **Jan-20** | Intercept | -3.646 | 0.7015 | -5.197 | 8.59E-07 | R^2^= 0.4184 F = 84.875 p = 1.459e-15 |
|  | Size | 1.3195 | 0.1432 | 9.213 | 1.46E-15 |  |
|  |  |  |  |  |  |  |
|  |  |  |  |  |  |  |
