# Supplementary material for: Revealing the status of Orbicella: Main reef-builder of Morrocoy National Park and Cuare Wildlife Refuge, Venezuela, Southern Caribbean
Source: PLoS One. 2025 Feb 7;20(2):e0317728. doi: 10.1371/journal.pone.0317728 (PMC11805429; doi:10.1371/journal.pone.0317728)
Supplement: Attachment — Submitted filename: Lee_PONE-D-24-34496_reviewer.pdf [file pone.0317728.s007.pdf]

PLOS ONE

Revealing the status of Orbicella: Main reef-builder of Morrocoy National Park and Cuare Wildlife Refuge, Venezuela, Southern Caribbean

--Manuscript Draft--

**Manuscript Number:**	PONE-D-24-34496
**Article Type:**	Research Article
**Full Title:**	Revealing the status of Orbicella: Main reef-builder of Morrocoy National Park and Cuare Wildlife Refuge, Venezuela, Southern Caribbean
**Short Title:**	Star corals status in two Marine Protected areas of Venezuela
**Corresponding Author:**	Anaurora Yranzo Duque<br>Central University of Venezuela: Universidad Central de Venezuela<br>Caracas, VENEZUELA, BOLIVARIAN REPUBLIC OF
**Keywords:**	coral reefs, colonies sizes, cover, density, mortality, Orbicella, southern Caribbean
**Abstract:**	Reef-building corals are the main basis of coral reef ecosystems, and the Orbicella genus is nowadays the most important in the Caribbean region. Although Orbicella species have been extensively studied, there are gaps in some southern Caribbean areas where there is a lack of baseline information that is crucial for management and conservation plans. In this study, we aimed to describe the population status of Orbicella faveolata and Orbicella annularis in two of the most important coastal marine protected areas of Venezuela: Morrocoy National Park and Cuare Wildlife Refuge. The study was carried out during 2018-2020, using the AGRRA Protocol in 16 reefs classified into five sectors. Among the two Orbicella species, O. faveolata was dominant in both MPA´s with higher densities and live cover, and only spatial and not temporal differences were found. Diseases were the primary threat to both species. Two out of the five sectors surveyed: the central sector of Morrocoy and reefs from Cuare are the most relevant for Orbicella populations, due to the higher live cover and abundance of reproductive colonies. Adequate MPA management is essential for the conservation of the Morrocoy-Cuare coral system, including the reduction of local anthropogenic stress sources, such as oil spills, uncontrolled tourism, and sewage discharges. The presence nowadays of the invasive exotic octocoral Unomia stolonifera in a study site reef (Cayo Sur-Cuare) is a threat that should be urgently addressed.
**Order of Authors:**	Anaurora Yranzo Duque
	Ana Teresa Herrera-Reveles
	Estrella Villamizar
	Francoise Cabada-Blanco
	Jeannette Pérez-Benítez
	Hazael Boadas
	José G Rodríguez-Quintal
	Carlos Pereira
	Samuel Narciso
	Freddy A. Bustillos
**Additional Information:**	
**Question**	**Response**
**Financial Disclosure**<br><br>Enter a financial disclosure statement that describes the sources of funding for the	Yes

Powered by Editorial Manager® and ProduXion Manager® from Aries Systems Corporation

work included in this submission. Review the submission guidelines for detailed requirements. View published research articles from *PLOS ONE* for specific examples.<br><br>This statement is required for submission and **will appear in the published article** if the submission is accepted. Please make sure it is accurate.<br><br>**Funded studies**<br>Enter a statement with the following details:<br>• Initials of the authors who received each award<br>• Grant numbers awarded to each author<br>• The full name of each funder<br>• URL of each funder website<br>• Did the sponsors or funders play any role in the study design, data collection and analysis, decision to publish, or preparation of the manuscript?<br><br>Did you receive funding for this work?	
Please add funding details.<br>as follow-up to "**Financial Disclosure**<br><br>Enter a financial disclosure statement that describes the sources of funding for the work included in this submission. Review the submission guidelines for detailed requirements. View published research articles from *PLOS ONE* for specific examples.<br><br>This statement is required for submission and **will appear in the published article** if the submission is accepted. Please make sure it is accurate.	AYD<br>WITHOUT NUMBER<br>EDGE of Existence Program of the Zoological Society of London-<br>https://www.edgeofexistence.org/blog/meet-new-orbicella-corals-fellow-ana-yranzo/<br>Clear Reef Social Fund- https://www.clear-reef.com/social_fund-objective_of_the_fund.php<br>- The Edge Fellowship program includes training for doing the project from wich the manuscript was done

**Funded studies**<br>Enter a statement with the following details:<br>• Initials of the authors who received each award<br>• Grant numbers awarded to each author<br>• The full name of each funder<br>• URL of each funder website<br>• Did the sponsors or funders play any role in the study design, data collection and analysis, decision to publish, or preparation of the manuscript?<br><br>Did you receive funding for this work?"	
Please select the country of your main research funder (please select carefully as in some cases this is used in fee calculation).<br>as follow-up to "**Financial Disclosure**<br><br>Enter a financial disclosure statement that describes the sources of funding for the work included in this submission. Review the submission guidelines for detailed requirements. View published research articles from *PLOS ONE* for specific examples.<br><br>This statement is required for submission and **will appear in the published article** if the submission is accepted. Please make sure it is accurate.<br><br>**Funded studies**<br>Enter a statement with the following details:<br>• Initials of the authors who received each award<br>• Grant numbers awarded to each author<br>• The full name of each funder<br>• URL of each funder website<br>• Did the sponsors or funders play any role in the study design, data collection and analysis, decision to publish, or preparation of the manuscript?<br><br>Did you receive funding for this work?"	UNITED KINGDOM - GB
**Competing Interests**	The authors have declared that no competing interests exist

Use the instructions below to enter a competing interest statement for this submission. On behalf of all authors, disclose any competing interests that could be perceived to bias this work—acknowledging all financial support and any other relevant financial or non-financial competing interests.<br><br>This statement is required for submission and **will appear in the published article** if the submission is accepted. Please make sure it is accurate and that any funding sources listed in your Funding Information later in the submission form are also declared in your Financial Disclosure statement.<br><br>View published research articles from *PLOS ONE* for specific examples.<br><br>**NO authors have competing interests**<br><br>Enter: *The authors have declared that no competing interests exist.*<br><br>**Authors with competing interests**<br><br>Enter competing interest details beginning with this statement:<br><br>*I have read the journal's policy and the authors of this manuscript have the following competing interests: [insert competing interests here]*<br><br>* typeset	
**Ethics Statement**<br><br>Enter an ethics statement for this submission. This statement is required if the study involved:<br><br>• Human participants<br>• Human specimens or tissue<br>• Vertebrate animals or cephalopods<br>• Vertebrate embryos or tissues	N/A

• Field research<br><br>Write "N/A" if the submission does not require an ethics statement.<br><br>General guidance is provided below. Consult the submission guidelines for detailed instructions. **Make sure that all information entered here is included in the Methods section of the manuscript.**	

Powered by Editorial Manager® and ProduXion Manager® from Aries Systems Corporation

**Format for specific study types**<br><br>**Human Subject Research (involving human participants and/or tissue)**<br>• Give the name of the institutional review board or ethics committee that approved the study<br>• Include the approval number and/or a statement indicating approval of this research<br>• Indicate the form of consent obtained (written/oral) or the reason that consent was not obtained (e.g. the data were analyzed anonymously)<br><br>**Animal Research (involving vertebrate animals, embryos or tissues)**<br>• Provide the name of the Institutional Animal Care and Use Committee (IACUC) or other relevant ethics board that reviewed the study protocol, and indicate whether they approved this research or granted a formal waiver of ethical approval<br>• Include an approval number if one was obtained<br>• If the study involved *non-human primates*, add *additional details* about animal welfare and steps taken to ameliorate suffering<br>• If anesthesia, euthanasia, or any kind of animal sacrifice is part of the study, include briefly which substances and/or methods were applied<br><br>**Field Research**<br><br>Include the following details if this study involves the collection of plant, animal, or other materials from a natural setting:<br>• Field permit number<br>• Name of the institution or relevant body that granted permission	
**Data Availability**<br><br>Authors are required to make all data underlying the findings described fully available, without restriction, and from the time of publication. PLOS allows rare exceptions to address legal and ethical concerns. See the PLOS Data Policy and FAQ for detailed information.	Yes - all data are fully available without restriction

A Data Availability Statement describing where the data can be found is required at submission. Your answers to this question constitute the Data Availability Statement and **will be published in the article**, if accepted.<br><br>**Important:** Stating 'data available on request from the author' is not sufficient. If your data are only available upon request, select 'No' for the first question and explain your exceptional situation in the text box.<br><br>Do the authors confirm that all data underlying the findings described in their manuscript are fully available without restriction?	
**Describe where the data may be found in full sentences. If you are copying our sample text, replace any instances of XXX with the appropriate details.**<br><br>• If the data are **held or will be held in a public repository**, include URLs, accession numbers or DOIs. If this information will only be available after acceptance, indicate this by ticking the box below. For example: *All XXX files are available from the XXX database (accession number(s) XXX, XXX.).*<br>• If the data are all contained **within the manuscript and/or Supporting Information files**, enter the following: *All relevant data are within the manuscript and its Supporting Information files.*<br>• If neither of these applies but you are able to provide **details of access elsewhere**, with or without limitations, please do so. For example:<br><br>*Data cannot be shared publicly because of [XXX]. Data are available from the XXX Institutional Data Access / Ethics Committee (contact via XXX) for researchers who meet the criteria for access to confidential data.*<br><br>*The data underlying the results presented in the study are available from (include the name of the third party*	All relevant data are within the manuscript and its Supporting Information files.

Powered by Editorial Manager® and ProduXion Manager® from Aries Systems Corporation

and contact information or URL).<br>• This text is appropriate if the data are owned by a third party and authors do not have permission to share the data.<br><br>* typeset	
Additional data availability information:	Tick here if the URLs/accession numbers/DOIs will be available only after acceptance of the manuscript for publication so that we can ensure their inclusion before publication.

Manuscript
Click here to access/download;Manuscript;MAIN DOCUMENT YRANZO ET AL -PLOS ONE.docx

Research Article

Full title: Revealing the status of *Orbicella*: Main reef-builder of Morrocoy National Park and Cuare Wildlife Refuge, Venezuela, Southern Caribbean

Short title: Star corals status in two Marine Protected areas of Venezuela

Anaurora Yranzo-Duque[1,2,5*], Ana Teresa Herrera-Reveles[3,5¶], Estrella Villamizar[1,5¶], Francoise Cabada-Blanco[4,2¶], Jeannette Pérez-Benítez[1,4&], Hazael Boadas[5&], José G. Rodríguez-Quintal[6,5&], Carlos Pereira[7&], Samuel Narciso[8,5&], Freddy A. Bustillos[9&]

[1] Laboratorio de Ecología de Sistemas Acuáticos, Línea de Investigación Ecosistemas Marino-Costeros, Centro de Ecología y Evolución, Instituto de Zoología y Ecología Tropical, Universidad Central de Venezuela. Caracas, Venezuela.

[2] EDGE of Existence Programme, Conservation and Policy, Zoological Society of London, London, UK.

[3] Laboratorio de Ecología Humana, Centro de Ecología Aplicada, Instituto de Zoología y Ecología Tropical. Universidad Central de Venezuela. Caracas, Venezuela.

[4] Institute of Marine Sciences, University of Portsmouth, Hampshire, UK

[5] Fundación Científica Los Roques (FCLR). Caracas, Venezuela

[6] Laboratorio de Biología Marino Costera (BioMaC-UC), Departamento de Biología-FACYT, Universidad de Carabobo. Valencia, Venezuela

[7] Laboratorio Taxonomía y Ecología de Algas y Macrofitas Marinas. Instituto de Biología Experimental, Universidad Central de Venezuela

[8] Fundación para la Defensa de la Naturaleza (FUDENA). Caracas, Venezuela.

[9] Gerencia de Ordenación Pesquera, Instituto Socialista para la Pesca y Acuicultura (INSOPESCA), Caracas, Venezuela

* Corresponding author E-mail: cursosayranzo@gmail.com (AYD)

Abstract

Reef-building corals are the main basis of coral reef ecosystems, and the *Orbicella* genus is nowadays the most important in the Caribbean region. Although *Orbicella* species have been extensively studied, there are gaps in some southern Caribbean areas where there is a lack of baseline information that is crucial for management and conservation plans. In this study, we aimed to describe the population status of *Orbicella faveolata* and *Orbicella annularis* in two of the most important coastal marine protected areas of Venezuela: Morrocoy National Park and Cuare Wildlife Refuge. The study was carried out during 2018-2020, using the Atlantic and Gulf Rapid Reef Assessment Protocol in 16 reefs classified into five sectors. Among the two *Orbicella* species, *O. faveolata* was dominant in both MPA´s with higher densities and live cover, and only spatial and not temporal differences were found. Diseases were the primary threat to both species. Two out of the five sectors surveyed: the central sector of Morrocoy and reefs from Cuare are the most relevant for *Orbicella* populations, due to the higher live cover and abundance of reproductive colonies. Adequate MPA management is essential for the conservation of the Morrocoy-Cuare coral system, including the reduction of local anthropogenic stress sources, such as oil spills, uncontrolled tourism, and sewage discharges. The presence nowadays of the invasive exotic octocoral *Unomia stolonifera* in a study site reef (Cayo Sur-Cuare) is a threat that should be urgently addressed.

Keywords: coral reefs, colonies sizes, cover, density, mortality, *Orbicella,* southern Caribbean

Introduction

The relevance of coral reefs around the globe is extensively documented (Woodhead et al., 2019; Santavy et al., 2021) as well as that of the coral species known as reef builders (Alvarez-Filip et al., 2009; Kuffner & Toth, 2016). These species are key for coral reef ecosystem functioning as they are essential to maintain the many benefits they provide to human populations (Edmunds, 2015). After the decline of *Acropora* species in the early 1980s (Aronson & Precht, 2001), *Orbicella* was considered the most important genus for shallow reef building corals in the Caribbean region (Roy, 2004). *Orbicella* species are distributed throughout over 30 countries in the Caribbean, Southern Florida, Bahamas, Bermuda, and Gulf of Mexico. Nevertheless, their populations, especially, *Orbicella annularis* and *Orbicella faveolata*, have experienced a drastic decline caused by bleaching

events, disease episodes, and other negative impacts caused by anthropic activities (Edmunds & Elahi, 2007; Bruckner & Hill, 2009). In general, their populations have been decreasing, with numerous studies reporting a reduction in coral cover and colony abundance (e.g., Edmunds, 2015; Van Woesik & Randall, 2017). Currently, *Orbicella annularis* and *Orbicella faveolata* are classified as endangered according to the last IUCN Red List Assessment (Rodríguez-Martínez et al., 2022). They are also included in CITES (Appendix II), the SPAW Protocol (Annex III), and in 2014, the National Marine Fisheries Services from the United States (NMFS) included them under the Endangered Species Act (ESA).

Implementing local conservation measures of any species specially considered endangered, requires the knowledge of its current local status. In Venezuela, a southern Caribbean country, information on *Orbicella* species is limited to some areas. The most recent publication dates back to 2015, which is part of the Red Book of Venezuelan Wildlife (National IUCN list), where only *O. annularis* was classified as Vulnerable and *O. faveolata* at the Least Concern category, according to a compilation of data prior to 2015 (Cróquer et al., 2015). In this study, we aimed at describing the population status of *Orbicella faveolata* and *Orbicella annularis* during 2018-2020, in two of the most important coastal marine protected areas of Venezuela: Morrocoy National Park and Cuare Wildlife Refuge. This study is the first focused on the ecology of *Orbicella* in this area of the southern Caribbean region.

Materials & methods

Study area

Morrocoy National Park and Cuare Wildlife Refuge are coastal marine protected areas, and the last is a RAMSAR site, in the central-western zone of Venezuela (Fig.1). Extensive mangrove forests, seagrass meadows, and coral reefs are present in both MPA´s (Almeida, 1976; Rodríguez, 1999). The livelihoods of the surrounding communities (such as Tucacas and Chichiriviche) depend directly on the MPAs ecosystems, as their main economic activities are tourism and artisanal fishing. However, restricted activities are established in the Zoning Management Plans.

In 1996, a massive mortality event of 90% of the benthic fauna of Morrocoy National Park occurred, including a drastic drop in coral cover (Laboy-Nieves et al., 2001). Chemical pollution or severe phytoplankton blooms followed by sudden oxygen depletion caused by climatic and oceanic anomalies, have been shown to be the main cause of this tipping point in the area (Rodríguez-Ramírez et al., 2008). In addition to this event, other continuous anthropogenic pressures derived from its proximity to the coast (Bastidas, Bone, & Garcia, 1999), affected the MNP, such as unregulated tourism, sediment and sewage discharges, and contamination from the oil industry that occurs in its vicinity (García *et al.,*2008, 2011). Unlike Morrocoy, coral reefs from Cuare were unaffected by the event, possibly due to current direction (Villamizar, 2008).

We assessed the following variables: density, cover, size structure and mortality in 16 reefs from five sectors (Fig. 1) which were selected based on a previous distribution assessment and considering to encompass the best spatial representation within the area. We use a modified version of the AGRRA Protocol 5.5 (Atlantic and Gulf Rapid Reef Assessment, Lang et al., 2012) from which a general benthos characterization was obtained. On each

reef, three 10 m^2 length transects were generally placed between 8 and 11 m depth. We did four temporal assessments (July and November 2018, July 2019, and late January 2020).

Data analysis

The data were analyzed using Primer V6. The main question to answer was whether there were spatial differences in the biological parameters of *Orbicella* (among sectors and reefs surveyed) and whether there were temporal differences (among survey periods). From this information, it was possible to identify the areas within the study site that were in the best condition and explore possible changes in the study period according to the survey date. The density, cover, and size of the colonies, as well as the percentages of mortality (recent, old, and total) were analyzed through analysis of variance (ANOVA), permutational analysis of variance (PERMANOVA) and SIMPER after testing assumptions. A contingency analysis (Pearson's chi-square) was used to assess the spatial and temporal differences in coral health status. Diseases were classified according to literature gross lesion descriptions (ej. Raymundo, Couch, & Harvell, 2008; Weil & Hooten, 2008).

For colony size, the recorded colonies had a minimum diameter of > 4 cm, as juveniles were ≤ 4 cm in size (Edmunds, Ross, & Didden, 2011). The size frequency distributions for both species were plotted. For *Orbicella annularis*, the Edmunds (2015) categories were used: size class I= ≤ 50 cm^2; size class II= > 50 cm^2- ≤150 cm^2; size class III= > 150 cm^2 - ≤ 250 cm^2; size class IV= > 250 cm^2. The size of the colonies was also plotted considering the reproductive size reported for each species: > 200 cm^2 area according to Szmant-Froelich (1985) for *Orbicella annularis* and > 100 cm maximum diameter for *Orbicella faveolata* (Porto *et al.,* 2015). Similarly, density analysis of reproductive and non-reproductive colonies was performed between sectors. In addition, a simple regression

analysis was performed to analyze the possible association between the percentage of total mortality and colony size.

Colonies were classified as alive (≤80% of partial mortality), almost dead (between >80-<100% of partial mortality), and dead (100% mortality-standing dead), following the criteria of Bruckner & Hill (2009).

Results

Among the recorded coral genera, *Orbicella* was the predominant one for all sectors surveyed (Fig.2), with the exception of the banks, where *Millepora* had a greater cover (34.81%). The live coral cover was generally dominated by *Orbicella faveolata*, for which significant differences were found between sectors but not temporal variations (Supplementary Table 1). The Banks and Southern sectors had the lowest cover ($t \geq 3.00$, $df= 4$, $p < 0.05$) and the highest were in the Refuge sector, but there were no significant differences between the central and northern sectors ($t \leq 2.93$, $df= 4$, $p = 0.10$). We found *Orbicella annularis* in both reefs from Cuare and in the southern and center sectors of Morrocoy. For this species, live cover was higher in Cuare than in the other sectors surveyed ($t \geq 3.00$; $df= 4$, $p < 0.05$, average cover=5.5%). Previous reports related to *Orbicella* live cover at the study site are summarized in Supplementary Table 2.

Concerning colony health, 85.88% of *O. faveolata* and 73.68% of *O.annulari*s colonies fell inside the alive category (≤ 80% of partial mortality). Average total mortality (Fig.3) varied between 15.11% ($\pm$ 23.42) in the Southern and 35.69% ($\pm$ 22.04) in the Refuge for *O. faveolata* and 37.08% ($\pm$32.01) to 50.42% ($\pm$22.00) in the Refuge and Center for *O. annularis*. Tissue mortality categories had some spatial and temporal differences (Supplementary Table 3). The old mortality stage was higher in relation to recent mortality, and reefs from the Southern sector had lower percentages of recent mortality compared to

reefs in the central sector (t = 3.68, df= 4, p = 0.02) and Refuge (t = 4.64, df= 4, p = 0.01). Average values of recent mortality were between 0.45% (Banks) to 12.97% (Center) and for all sectors, recent mortality was higher in the last survey (late January 2020) than in the first one in July 2018 (t = 3.53, df= 3, p = 0.0083). For *O. annularis* there were not spatial differences in mortality categories (PERMANOVA; recent mortality, F= 0,094729, df=3, p = 0.9599; old mortality; F= 1,0218, df=3, p = 0.4573) and the average value of recent mortality for all surveys was 1.81%.

The main detrimental factor for both species were the diseases, being Yellow Band Disease the most frequent observed in *Orbicella faveolata* (Fig. 4). The density of diseased *O. faveolata* colonies varied among the reefs (X^2 = 30.169, df=4, p = 0.0015), being lower in the Southern sector. The average prevalence of diseases varied between 2.78% in the northern (Borracho reef) and 55% in the Center (Caiman reef). We recorded the highest values mainly at the Center and Refuge, with an average of 25.66% (± 28.64) and 24.32% (± 22.49), respectively. In general, prevalence was highest in July and January had less diseased colonies. However, the temporal density of the diseased colonies was not significantly different (X^2 = 3.425, df = 2, p = 0.1804). We found three diseases in *O. annularis*: White Plague Disease, Yellow Band disease, and Dark Spot Disease (Fig. 4E-G) without differences in density of diseased colonies between reefs and sectors (X^2 = 4.672, df=4, p = 0.587). We observed an additional health condition mostly in *Orbicella faveolata* colonies, a dark line bordering the live tissue adjoining the dead area (Fig.4H). Other detrimental health factors we identified at a lower frequency were bioerosion by *Cliona* sponges, grazing by damselfish (Pomacentridae), bleaching and mechanical damage.

With respect to size, of the total alive *O. faveolata* colonies we measured, 42.12% were of adult reproductive size (more than 100 cm maximum diameter, Fig. 5 above). The density of *O. faveolata* reproductive colonies varied among sectors (Supplementary Table 4) and was higher in the Refuge ($t \geq 4.23$, df= 4, $p \leq 0.022$), except for the Center ($t = 1.63$, df= 4, $p = 0.20$), on which a high proportion of adult colonies was also found. Meanwhile, the Banks had densities significantly lower than those of the Northern, Central, and Refuge sectors ($t \geq 2.95$, df= 4, $p \leq 0.05$) and a density similar to that of the Southern sector ($t = 1.44$, df= 4, $p = 0.24$). With regard to *Orbicella annularis*, most of colonies were in size class IV (max. diameter = >250 cm^2, Fig. 5 below). For this species, 53.84 % of colonies had a reproductive size and we found them mainly in Cuare (85.71%). No *Orbicella* recruits were observed during the surveys.

Finally, regarding colony size and mortality, we found a positive relationship for *O. faveolata*, with average mortality increasing in size classes with larger colonies (Supplementary Table 5), although the strength of the correlation was low, ranging between 0.06 and 0.192. In contrast, *Orbicella annularis* colony size and total mortality were not related (ANOVA, F=0.27; p=0.61).

Discussion

We assessed the status of the main coral reef builders, *Orbicella annularis* and *O. faveolata*, in Morrocoy National Park and Cuare Wildlife Refuge in Venezuela, southern Caribbean. The results indicated that *Orbicella faveolata* is the dominant species for both MPAs coral reefs. Among the sectors we evaluated, the Center in Morrocoy and the Refuge in Cuare, were the most relevant for *Orbicellas´*s population because of the higher live cover and density of reproductive colonies. The reefs where *Orbicella* colonies are in better health condition nowadays, are the same healthier more than 20 years ago:

Sombrero at the Center sector (the less affected by the massive die off due to the intense currents which also diminish the continue sediment discharges and freshwater influence from local rivers) and Cayo Norte in Cuare, unaffected by the massive mortality (Laboy-Nieves et al., 2001; Bone et al., 2001; Villamizar, 2008). However, tissue mortality caused by detrimental factors among which diseases can be highlighted, is a concern for the future of this species, even when at the time of this study it was not observed the most recent coral disease reported: Stony Coral Tissue Loss disease (SCTLD) for which *Orbicella* species have been categorized as intermediately susceptible or highly susceptible (Kramer et al., 2022).

The dominance of *Orbicella* species before the mass mortality event (1996) has been reported for some reefs along the MNP, but data were limited to live cover (see Table S2 for references) which can be a poor indicator of the abundance of a coral species, unlike colony density (especially Orbicella, Edmunds & Elahi, 2007). Differences in depth surveys, as well as cover estimation methods (ej. use of quadrats, chain transects) makes it difficult to obtain a clear trend over time for *Orbicella* species. Nevertheless, taking them as a reference, reefs of MNP had a decrease in *O. annularis* cover in most reefs. In the case of *O. faveolata*, there are no cover records before the massive die off; however, considering only the last previous records (years 2004-2005), the average cover estimations for the present study are similar for some reefs (Villamizar, 2008). More recently (2017-2018), Miyazawa et al., (2020) reported the dominance of *O. faveolata* in Caiman and Sombrero (center sector) and the three coral cays of the refuge (Norte, Sur and Medio) in 2017-2018.

General trends of *Orbicella* species in the Caribbean region are a decline in cover and abundance (Bruckner & Bruckner, 2006; Edmunds & Elahi, 2007; Edmunds et al., 2014). However, a status review (Moulding & Ladd, 2022) indicates a variable situation with a mixed population trends of some decline and some stability due to the common presence of colonies in some sites (e.g., Florida, Puerto Rico, US Virgin Islands) but scarce in others (e.g., Cuba). Unlike many Caribbean reefs (e.g., González-Díaz et al., 2018; Caballero Aragón et al., 2019), *Orbicella* species at the study site do not appear to have been replaced by weedy/opportunistic corals, only the most deteriorated sites, e.g., the Southern sector had a higher abundance of weedy corals than *Orbicella* (e.g., *Porites*).

Regarding *Orbicella* colony size, the proportion of adult colonies can indicate their reproductive potential, although it has been pointed out that the effective population size may be smaller than the total standing population, because of clonality and low reproductive compatibility (Miller et al., 2018). Furthermore, the finding of a positive relationship between size and mortality for *O. faveolata* colonies, can compromise the reproduction scope of this species, as larger colonies use to have highest partial mortality, which is common due to more time exposed to different disturbances (Hughes & Jackson, 1980; Meesters et al., 1996; Meesters et al., 1997) and the loss of living tissue affects the ability of corals to reproduce sexually (Roy, 2004). Smaller colonies in the Southern sector with less mortality, are far away from a healthier condition because they are probably a result of colony fission by partial mortality (Hughes & Tanner, 2000). Besides, high levels of recent mortality as some we obtained is indicative of conditions in which growth and regeneration rates are not able to keep up with mortality, and suggests that a disturbance is ongoing (Bruckner, 2012; Lirman, 2014) which increased during the last survey, with ´´red flags´´ values above 5% (Healthy Reefs, 2008).

During the present study, no *Orbicella* recruits were seen. The only recruit's species observed were fast-growing species from the genus *Agaricia*, *Porites* and *Pseudodiploria*, likewise to that reported by Villamizar (2008). The survey period has been pointed out as an important variable to detect *Orbicella* recruits, because in the usual duration of projects research, the low recruitment that characterizes this species hinders detection of recruits (Edmunds & Elahi, 2007; Edmunds, 2015). According to Edmunds *et al.* (2011) the low recruitment of *Orbicella* has been related to high post-settlement mortality or low abundance of settlement cues from coralline algae as was pointed out for *Acropora* (Ritson-Williams *et al*. 2010) and not to failed reproduction or poor fertilization success (Szmant 1991; Levitan et al. 2004).This highlights the importance of doing long-term studies in long-lived animals like *Orbicella*.

Mortality factors found for *Orbicella* species in the study site were similar to numerous studies conducted around the Caribbean region, including Los Roques National Park in Venezuela, with Yellow Band Disease (YBD) and White Plague Disease (WPD) as the most important diseases (García, Cróquer, & Pauls, 2003; Sutherland *et al*., 2004; Bruckner & Bruckner 2006; Villamizar *et al.,*2014; Weil & Rogers 2011; Williams *et al.,* 2020). *Orbicella*'s health assessment has allowed us to obtain a broader vision of this topic as previous records of diseases from the study area were limited to Sombrero in the MNP and Cayo Norte in Cuare (Ramos-Flores, 1983; Weil, Urreiztieta, & Garzón-Ferreira, 2000; Croquer & Bone, 2003; Cróquer, Bastidas, & Lipscomb, 2006; Montilla *et al.,* 2016). It is important to highlight that histopathology examination of diseased colonies should be done to have robust data on this topic in the study area (Work, Aeby, & Hughen, 2016; Price & Peters, 2018). With respect to the finding of the condition described as Thin Dark Line Syndrome (TDL), an assessment over time must be performed as it could be a sign of

changes in the microbiome or in the adjacent bacterial biota of the coral (E. Jordan comm pers), although it may also be a pigmentation response to the presence of algal mats and sediments (Rodríguez-Martínez comm pers). Spatial differences obtained for the density of diseased colonies are probably related to density-dependent processes (Lafferty & Holt, 2003) as the reefs with fewer diseased colonies had the lowest *Orbicella* abundance.

Important local stress factors not addressed like overfishing, sediment and sewage discharges, pollution including oil spills, coastal development, and associated water quality degradation are important threats in the study area, which can affect the *Orbicella*´s life cycle, since larval settlement (Jovanovic & Guzman, 2014; Sabine et al., 2015; Oliver et al., 2018). Reduction of local threats is essential, as it is well known that stress factors can affect the immune coral system (Carilli et al., 2009; Dougan et al., 2020). For example, Montilla et al. (2016) studied the effects of hydrocarbon pollution on healthy and diseased colonies of *O. faveolata* from Sombrero reef and found reduced enzymatic activity in colonies infected with Yellow Band Disease (the most frequent disease recorded in the present study) suggesting that diseased colonies may be more vulnerable to the effects of chemical pollution. The recurrent oil spills that occurred in recent years, originating from the oil refinery located near both MPA´s, can exacerbate this situation. Other significant threats in the area are the exotic octocoral *Unomia stolonifera* introduced by acuarists since 2007 on the east coast of Venezuela (Ruiz-Allais et al., 2014) currently present in Cuare (Cayo Sur) as well as the temperature increase because of climate change projections.

In conclusion, special attention must be drawn to reefs from the Cuare Wildlife Refuge and the central sector of Morrocoy National Park for the conservation of the *Orbicella*

population and, therefore, of the good and services coral reefs provide in the area. Proper management by authorities, including the control of human activities, is critical for the future of these coral communities. Maintaining and complying with the Cuare reef access restriction established by law, its fundamental and responsible and sustainable tourism must be promoted along all Morrocoy National Park, but especially in the central sector. The tourists and other national park users must be included in awareness campaigns to improve the effectiveness of the control and surveillance actions.

Acknowledgements. Thanks to the EDGE family for the guidance, mentoring and training during the EDGE of Existence program fellowship, especially to Olivia, Claudia, Davi, Fran, Charlie and Cassandra. Thank you to Idea Wild for the donation of essential equipment for the development of the Project and FONPESCA for the motorboat oil donation. This study would not have been possible without the teamwork of all participants.

References

Alvarez-Filip, L., Dulvy, N. K., Gill, J. A., Côté, I. M. & Watkinson, A. R. (2009). Flattening of Caribbean coral reefs: region-wide declines in architectural complexity. *Proceedings of the Royal Society. B*, 276, 3019-3025.

Aronson, R.B. & Precht, W.F. (2001). White-band disease and the changing face of Caribbean coral reefs. *Hydrobiologia,* 460, 25–38.

Bone, D., Cróquer, A., Klein, E., Pérez, D., Losada, F., Martin, A., Bastidas, C., Rada, M., Galindo, L. & Penchaszadeh, P. (2001). Programa Caricomp: Monitoreo a largo plazo de los ecosistemas marinos del Parque Nacional Morrocoy, Venezuela. *Interciencia,* 26, 457-462.

Bruckner, A. W. & Bruckner, R. (2006). The recent decline of *Montastraea annularis* (complex) coral populations in western Curaçao: a cause for concern? *Revista de Biología Tropical,* 54, 45-58.

Bruckner, A. W. & Hill, R. (2009). Ten years of change to coral communities off Mona and Desecheo Islands, Puerto Rico from disease and bleaching. *Disease of Aquatic Organisms*, 87, 19–31.

Bruckner, A. W. (2012). Static measurements of the resilience of Caribbean coral populations. *Revista de Biología Tropical,* 60, 39-57.

Caballero Aragón, H., Armenteros, M., Perera Valderrama, S., Rey Villiers, N., Cobián Rojas, D., Campos Verdecia, K. & Alcolado Menéndez, P. M. (2019). Ecological condition of coral reef assemblages in the Cuban Archipelago. *Marine Biology Research,* 15, 61-73.

Carilli, J. E., Norris, R. D., Black, B. A., Walsh, S. M. & McField, M. (2009). Local stressors reduce coral resilience to bleaching. *Plos one*, 4, e6324.

Cróquer, A., Villamizar, E., Yranzo, A., Zubillaga, A.L. & Bastidas, C. (2015). Coral pilar Estrella *Orbicella annularis*. In: J.P. Rodríguez, A. Garcìa-Rawlins & F. Rojas-Suarez (eds). Libro Rojo de la Fauna Venezolana. Cuarta Edición. Provita y Fundación Empresas Polar, Caracas, Venezuela.

Dougan, K.E., Ladd, M.C., Fuchs, C., Vega Thurber, R., Burkepile, D.E. & Rodriguez-Lanetty, M. (2020). Nutrient pollution and predation differentially affect innate immune pathways in the coral *Porites porites. Frontiers of Marine Science*, 7, 563865.

Edmunds, P. J. (2015). A quarter-century demographic analysis of the Caribbean coral, *Orbicella annularis*, and projections of population size over the next century. *Limnology and Oceanography*, 60, 840–855.

Edmunds, P. J., Pochon, X., Levitan, D. R., Yost, D. M., Belcaid, M., Putnam, H. M. & Gates, R.D. (2014). Long-term changes in *Symbiodinium* communities in *Orbicella annularis* in St. John, US Virgin Islands. *Marine Ecology Progress Series,* 506, 129-144.

Edmunds, P.J., Ross, C.L.M. & Didden, C. (2011). High, but localized recruitment of *Montastraea annularis* complex in St. John, United States Virgin Islands. *Coral Reefs* 30: 123–130.

Edmunds, P. & Elahi, R. (2007). The demographics of a 15-years decline in cover of the Caribbean reef coral *Montastraea annularis*. *Ecological Monographs*, 77, 3-18.

González-Díaz, P., González-Sansón, G., Aguilar Betancourt, C., Álvarez Fernández, S., Perera Pérez, O., Hernández Fernández, L., Ferrer Rodríguez, V.M., Cabrales Caballero, Y., Armenteros Almanza, M & de la Guardia Llanso, E. (2018). Status of Cuban coral reefs. *Bulletin of Marine Science,* 94, 229-247.

Healthy reefs for Healthy people (2008). Quick reference guide 2008. A companion to a Guide to indicators of reef health and social well-being in the Mesoamerican reef region. 28 pp.

Hughes TP & Jackson JBC (1980). Do corals lie about their age? Some demographic consequences of partial mortality, fission, and fusion*. Science,* 209, 713−715.

Hughes, T.P. & Tanner, J.E. (2000). Recruitment Failure, Life Histories, and Long-Term Decline of Caribbean Corals. *Ecology*, 81, 2250-2263.

Jovanovic, B. & Guzman, H. M. (2014). Effects of titanium dioxide (TiO2) nanoparticles on caribbean reef-building coral (*Montastraea faveolata*). *Environmental Toxicology and Chemistry*, 33, 1346-53.

Kuffner, I. B.& Toth, L. T. (2016). A geological perspective on the degradation and conservation of western Atlantic coral reefs. *Conservation Biology*, 30, 706-715.

Kramer, P.R., Kramer, J., Roth, L., & D. Larios. (2022). Field guide to monitoring coral disease outbreaks in the Mesoamerican Region. Integrated Ridge to Reef Management of the Mesoamerican Reef Ecoregion Project (MAR2R-CCAD/GEF-WWF and MAR Fund). Guatemala City, Guatemala.

Laboy-Nieves, E., Klein, E., Conde, J., Losada, F., Cruz, J. J. & Bone, D. (2001). Mass mortality of Tropical Marine Communities in Morrocoy, Venezuela. *Bulletin of Marine Science*, 68, 163–179.

Lang, JC., Marks, KW., Kramer, PR., Kramer, PA & Ginsburg, RN. (2012). AGRRA protocol version 5.5.

Lirman, D., Formel, N., Schopmeyer, S., Ault, J. S., Smith, S. G., Gilliam, D. & Riegl, B. (2014). Percent recent mortality (PRM) of stony corals as an ecological indicator of coral reef condition. *Ecological Indicators,* 44, 120-127.

Meesters, E. H., Wesseling, I. & Bak, R. P. (1996). Partial mortality in three species of reef-building corals and the relation with colony morphology. *Bulletin of Marine Science,* 58, 838-852.

Meesters, E. H., Wesseling, I. & Bak, R. P. (1997). Coral colony tissue damage in six species of reef-building corals: partial mortality in relation with depth and surface area. *Journal of Sea Research*, 37, 131-144.

Miller, M. W., Baums, I. B., Pausch, R. E., Bright, A. J., Cameron, C. M., Williams, D. E., ... & Woodley, C. M. (2018). Clonal structure and variable fertilization success in Florida Keys broadcast-spawning corals. *Coral Reefs,* 37, 239-249.

Montilla, L. M., Ramos, R., García, E. & Cróquer, A. (2016). Caribbean yellow band disease compromises the activity of catalase and glutathione S-transferase in the reef-building coral *Orbicella faveolata* exposed to anthracene. *Disease of Aquatic Organisms*, 119, 153-161.

Moulding, A. & Ladd, M. (2022). Staghorn coral (*Acropora cervicornis)*, elkhorn coral (*Acropora palmata*), lobed star coral *(Orbicella annularis*), mountainous star coral (*Orbicella faveolata*), boulder star coral (*Orbicella franksi*), rough cactus coral (*Mycetophyllia ferox*), and pillar coral (*Dendrogyra cylindrus*) 5-year review. Summary and Evaluation. United States. National Marine Fisheries Service.

Oliver, L. M., Fisher, W. S., Fore, L., Smith, A. & Bradley, P. (2018). Assessing land use, sedimentation, and water quality stressors as predictors of coral reef condition in St. Thomas, US Virgin Islands. *Environmental Monitoring Assessment*, 190, 1-16.

Porto-Hannes, I., Zubillaga, A. L., Shearer, T. L., Bastidas, C., Salazar, C., Coffroth, M. A. & Szmant, A. M. (2015). Population structure of the corals *Orbicella faveolata* and Acropora palmata in the Mesoamerican Barrier Reef System with comparisons over Caribbean basin-wide spatial scale. *Marine Biology,* 162, 81-98.

Raymundo, L.J., Couch, C.S. & Harvell, C.D (eds). (2008) Coral disease handbook: guidelines for assessment, monitoring and management. Coral Reef Targeted Research and Capacity Building for Management Program, Brisbane, Australia, ISBN: 978-1-9213-17-01-9.

Rodríguez-Martínez, R., Hoeksema, B., Rivera-Sosa, A., Villamizar, E., Vermeij, M., Croquer, A. & Banaszak, A. (2022). *Orbicella faveolata.* The IUCN Red List of Threatened Species 2022: e.T133373A165855828. Accessed on 02 July 2024.

Rodríguez-Ramírez, A., Bastidas, C., Rodríguez, S., Leão, Z., Kikuchi, R., Oliveira, M. et al. (2008). The Effects of Coral Bleaching in Southern Tropical America: Brazil, Colombia, and Venezuela. Status of Caribbean Coral Reefs after Bleaching and Hurricanes in 2005. Eds C. Wilkinson & D. Souter. Townsville, Australia: Global Coral Reef Monitoring Network, and Reef and Rainforest Research Centre.105-114.

Roy, R.E. (2004). Turf Algal/Sediment (TAS) Mats: A chronic stressor on scleractinian corals in Akumal, México. Doctoral thesis. University of Texas, Texas, USA.

Ruiz Allais, J. P., Amaro, M. E., McFadden, C. S., Halász, A. & Benayahu, Y. (2014). The first incidence of an alien soft coral of the family Xeniidae in the Caribbean, an invasion in eastern Venezuelan coral communities. *Coral Reefs*, 33, 287-287.

Sabine, A. M., Smith, T. B., Williams, D. E. & Brandt, M. E. (2015). Environmental conditions influence tissue regeneration rates in scleractinian corals. *Marine Pollution Bulletin*, 95, 253-264.

Santavy, D. L., Horstmann, C. L., Sharpe, L. M., Yee, S. H., & Ringold, P. (2021). What is it about coral reefs? Translation of ecosystem goods and services relevant to people and their well-being. *Ecosphere*, 12, e03639.

Szmant-Froelich, A. (1985). The effect of colony size on the reproductive ability of the Caribbean coral *Montastrea annularis* (Ellis and Solander). *In Proc 5th International Coral Reef Symposium* (Vol. 4, pp. 295-300).

Van Woesik, R. & Ryall, C.J. (2017). Coral disease hotspots in the Caribbean. *Ecosphere,* 8, e01814.

Villamizar, E. (2008). Status de los arrecifes coralinos del Parque Nacional Morrocoy, Sistemas degradados o en recuperación? Trabajo de Ascenso, Facultad de Ciencias, Universidad Central de Venezuela. 135 pp.

Woodhead A.J., Hicks, C.C., Norström, A.V., Williams, G.J., Graham, N.A. (2019). Coral reef ecosystem services in the Anthropocene. *Functional Ecology,* 33,1023–34.

Supporting information

S1 Table 1.

S1 Table 2.

S1 Table 3.

S1 Table 4.

S1 Table 5.

Figure
Click here to access/download;Figure;FIGURES AYRANZO PLOS ONE.docx

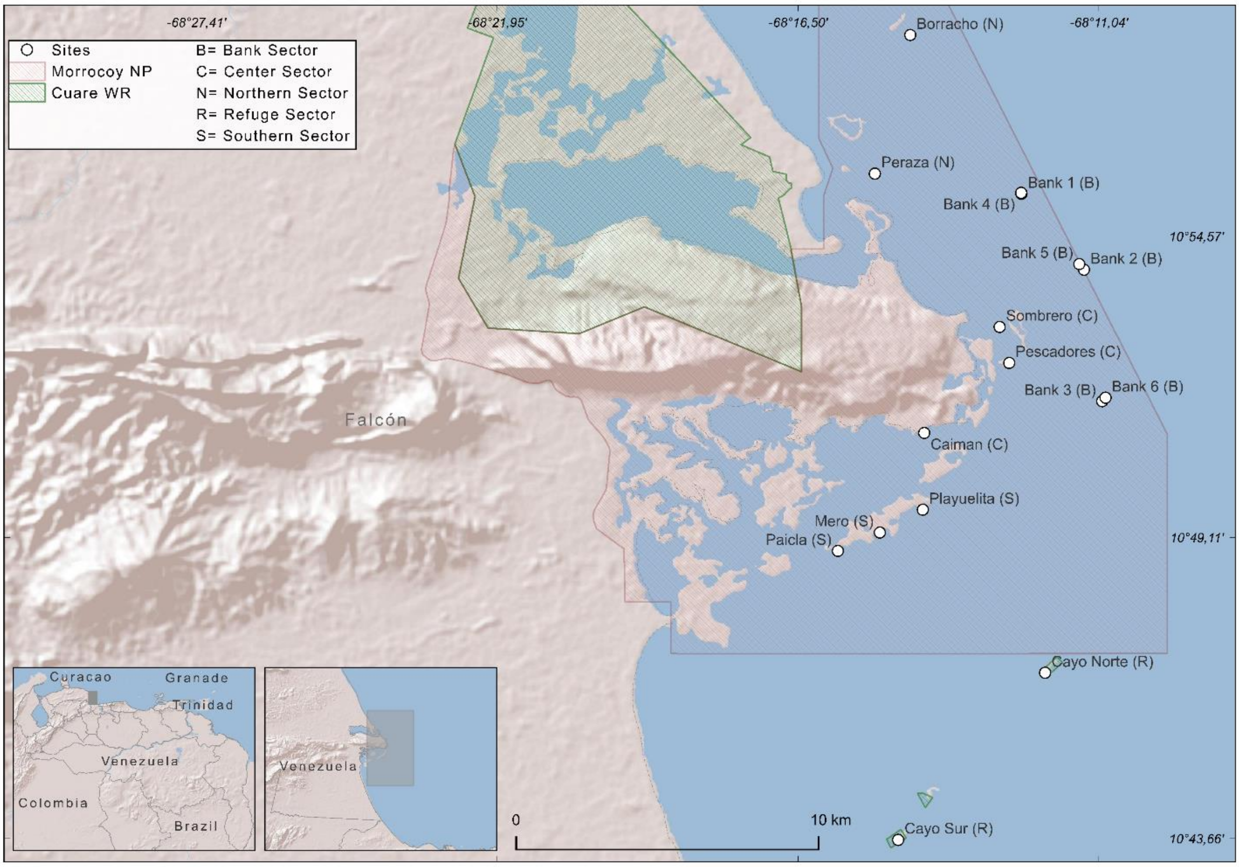

Fig. 1 Study sites in Morrocoy National Park and Cuare Wildlife Refuge, Falcon State, Venezuela- Southern Caribbean. Reefs assessed (n=16) were classified in five sectors denoted with letters in parenthesis on each reef name. All sites were fringing reefs except the banks which were areas dominated by branched octocorals and some sponges as well as isolated corals, forming mosaics with sandy bottoms, patches of coral rubble and rocky slabs.

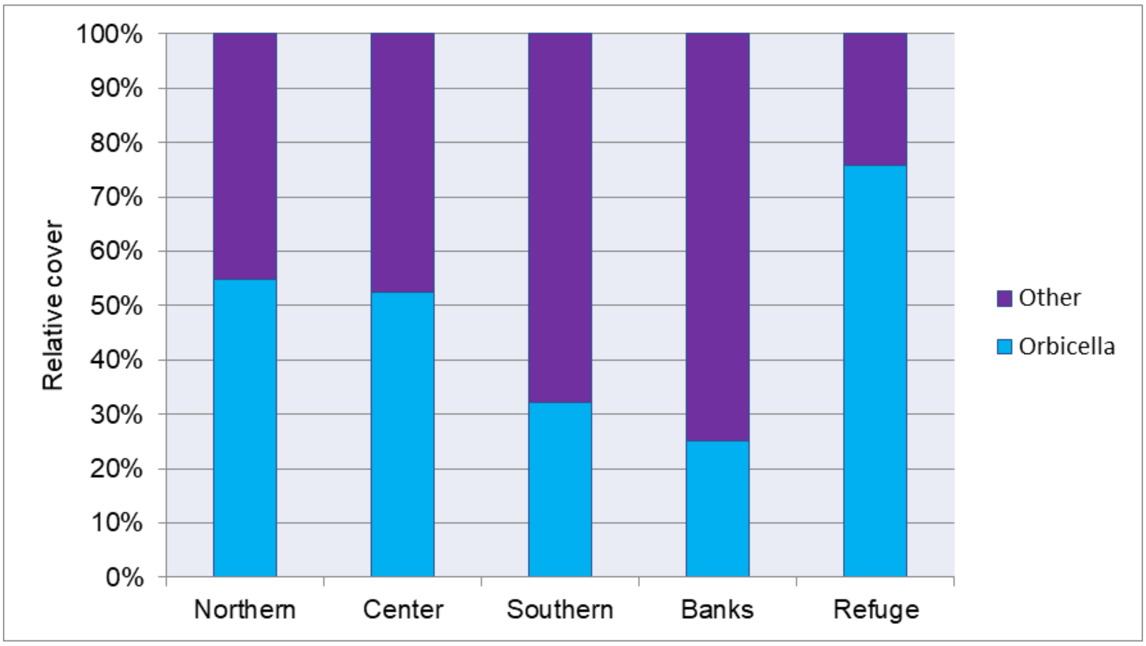

Figure 2. Relative cover (%) of *Orbicella* (*O. annularis* and O. *faveolata*) in Morrocoy National Park (Northern, Center, Southern, Bank sectors) and Cuare Wildlife Refuge (Refuge) in 2018-2020, Venezuela, southern Caribbean.

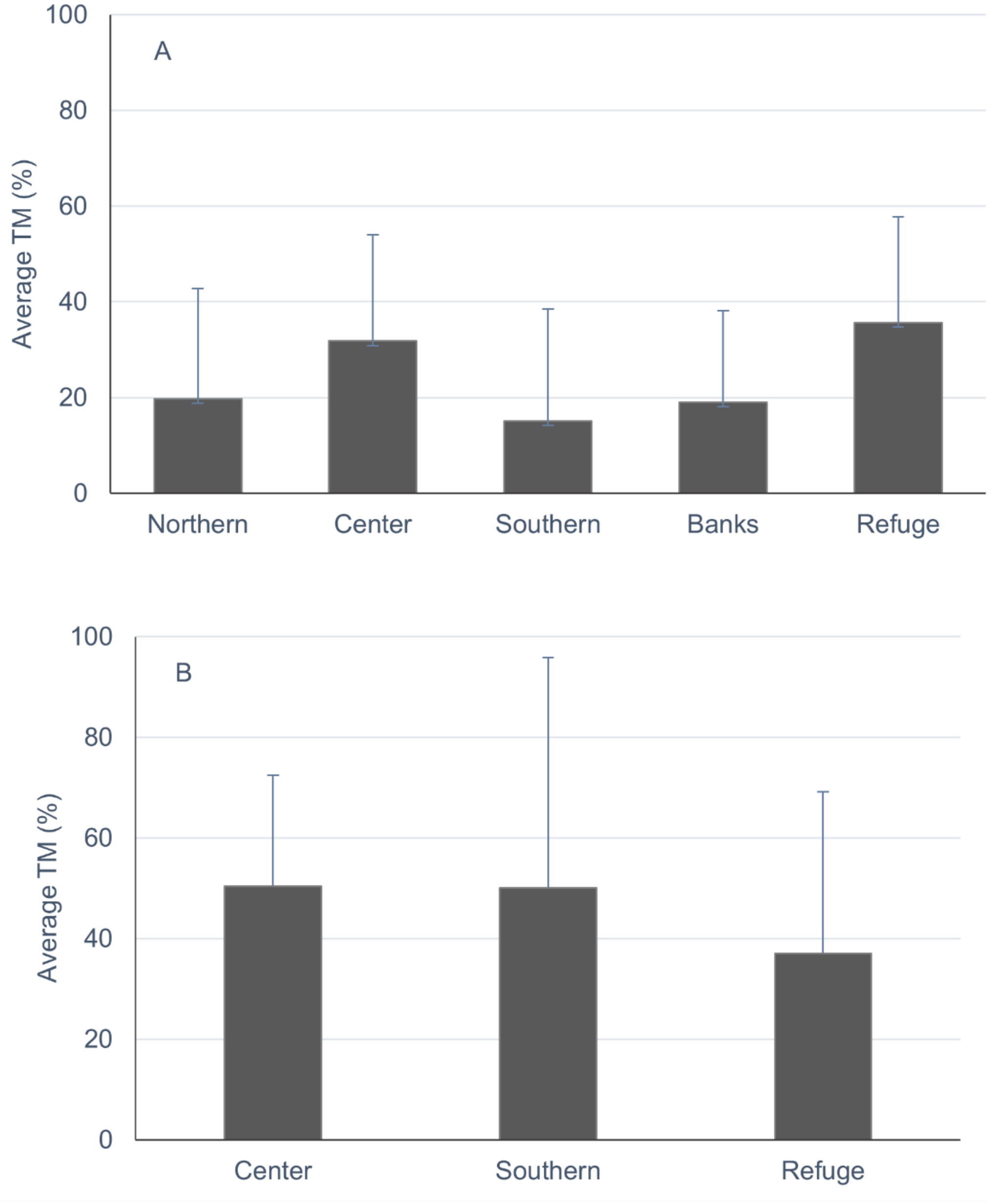

Figure 3. Average total mortality (TM) of *O. faveolata* (A) and *O. annularis* alive colonies (B) in Morrocoy National Park (Northern, Center, Southern and Banks) and Cuare Wildlife Refuge (Venezuela), in 10 m^2 transects, in 2018-2020. Bars are standard deviation (SD). Alive colonies = colonies with ≤80% partial mortality.

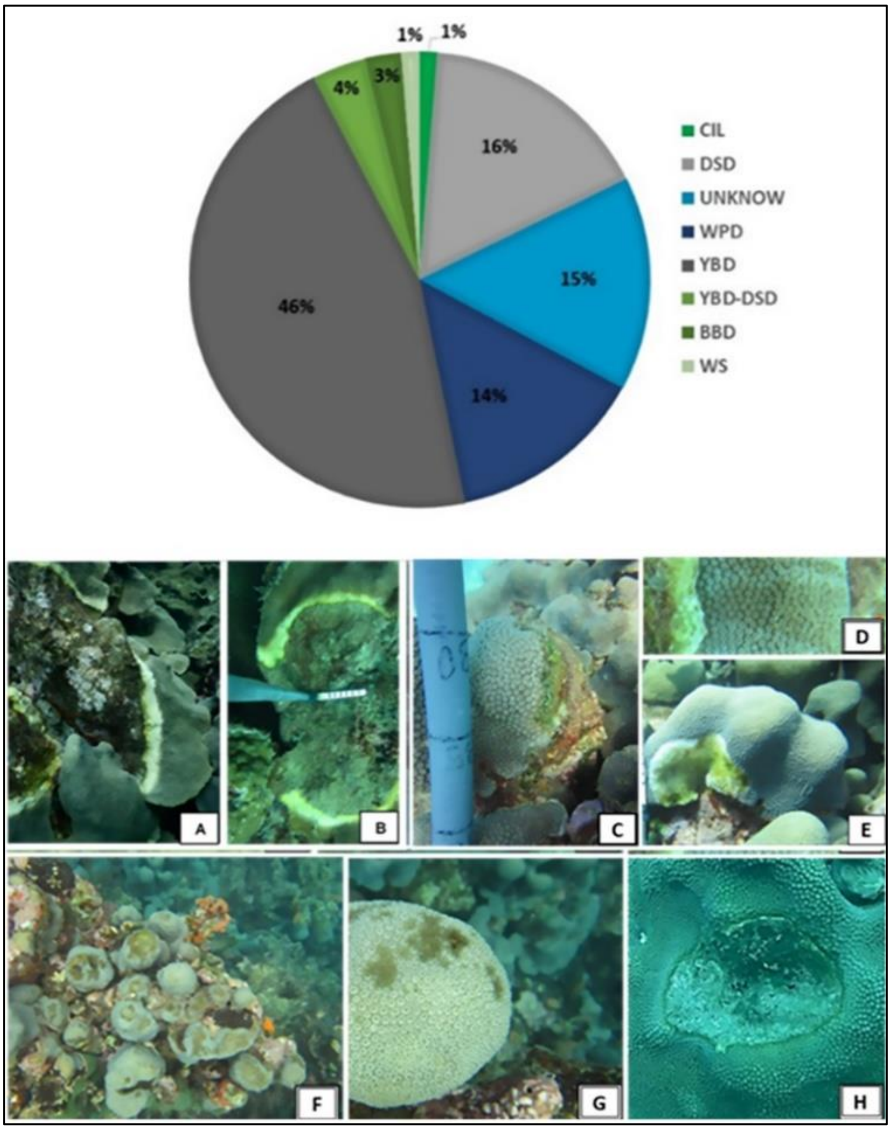

Figure 4. *Orbicella* diseases and syndromes in Morrocoy National Park and Cuare Wildlife Refuge, in 2018-2020, Venezuela. Graph showing diseases and syndromes in *O. faveolata*: CIL: Ciliate, DSD: Dark Spot Disease; WPD: White Plague Disease; YBD: Yellow Band Disease; BBD: Black Band Disease; WS: White Syndrome. Photos above: *Orbicella faveolata* with Yellow Band Disease (A-B); ciliate infection (C) and White plague disease (D); *Orbicella annularis* with White Plague Disease (E), Photos below: *O. annularis* with Dark Spot Disease (F-G) and Thin Dark Line in *O. faveolata* (H).

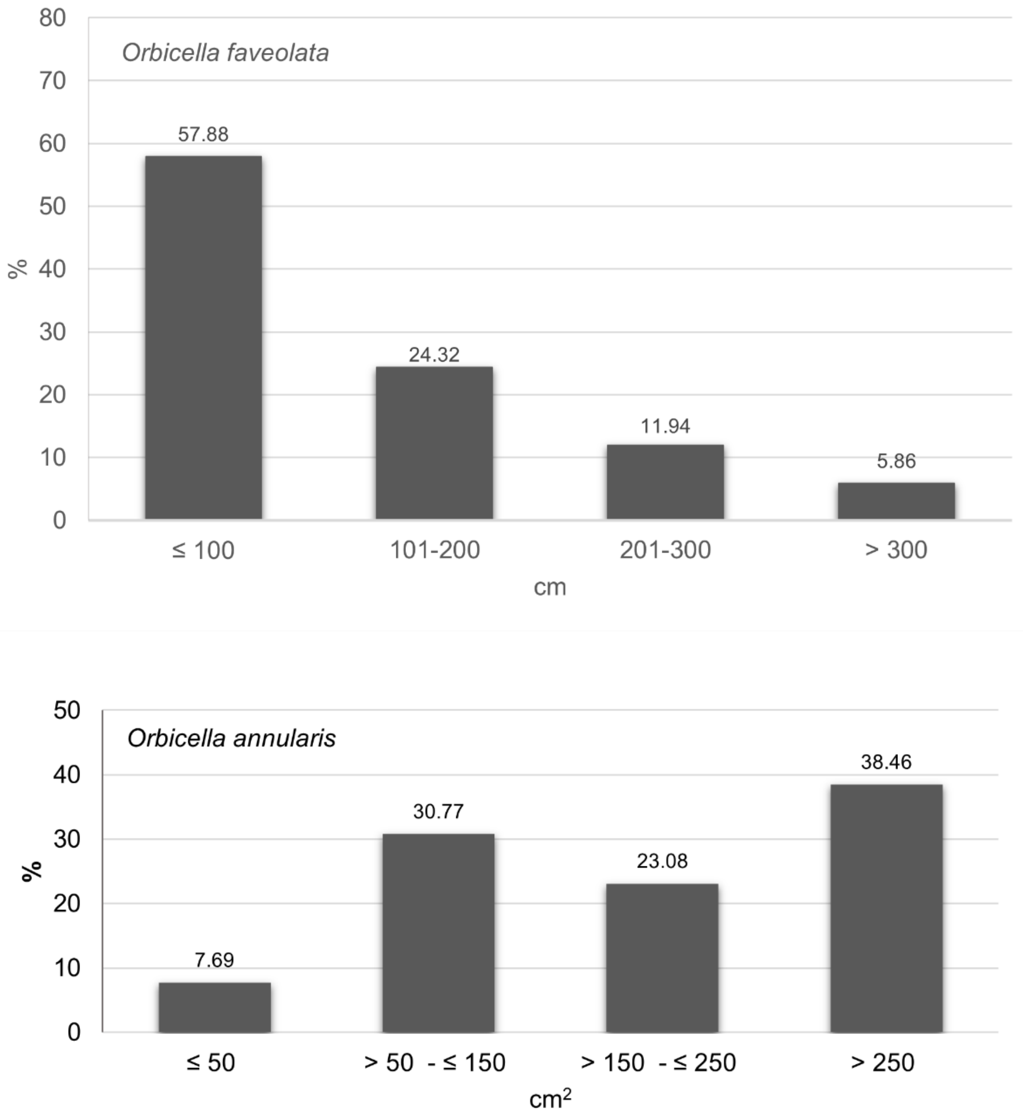

Figure 5. Size classes in *Orbicella faveolata* (above, N= 444 colonies with measures) and *Orbicella annularis* (below, N=13 colonies with measures) from Morrocoy National Park and Cuare Wildlife Refuge (Venezuela) in 10 m^2 transects, in 2018-2020. *O. faveolata* size classes according to adult reproductive sizes.

Suplementary tables

Click here to access/download
Supporting Information
SUPLEMENTARY TABLES-YRANZO ET AL.docx
