# Supplementary material for: Revealing the status of Orbicella: Main reef-builder of Morrocoy National Park and Cuare Wildlife Refuge, Venezuela, Southern Caribbean
Source: PLoS One. 2025 Feb 7;20(2):e0317728. doi: 10.1371/journal.pone.0317728 (PMC11805429; doi:10.1371/journal.pone.0317728)
Supplement: Attachment — Submitted filename: Response to reviewers.docx [file pone.0317728.s010.docx]

**Rebuttal letter**

We would like to thank the academic editor and the reviewers for their valuable comments of our manuscript to improve it.

All the comments were taking into account and we provide responses in this letter (bold font)

Sincerely,

**Anaurora Yranzo Duque**

**Corresponding author (on behalf of all co-authors),**

**To the academic editor**

Journal Requirements:

1.PLOS ONE's style requirements,

**Yes, all the format requirements were incorporated**

2. In your Methods section, please provide additional information regarding the permits you obtained for the work.

**A paragraph related with permits was included**

3. Please state what role the funders took in the study.

**The funders had no role in study design, data collection and analysis, decision to publish, or preparation of the manuscript.**

4. When completing the data availability statement of the submission form, you indicated that you will make your data available on acceptance.

**Yes, now data related to the study was included as a Supporting Information file following the principles of data in Supporting Information files**

5. Please amend either the abstract on the online submission form (via Edit Submission) or the abstract in the manuscript so that they are identical.

**Yes, already done**

6. Figure 1. We require you to either present written permission from the copyright holder to publish these figures specifically under the CC BY 4.0 license, or remove the figures from your submission: supply a replacement figure that complies with the CC BY 4.0 license

**The figure 1 was replaced by a base map elaborated with data from OpenStreetMap** **and OpenStreetMap Foundation, which is made available under the Open Database License**

7. Please review your reference list to ensure that it is complete and correct. If you have cited papers that have been retracted, please include the rationale for doing so in the manuscript text, or remove these references and replace them with relevant current references. Any changes to the reference list should be mentioned in the rebuttal letter that accompanies your revised manuscript. If you need to cite a retracted article, indicate the article’s retracted status in the References list and also include a citation and full reference for the retraction notice

**References changes have been made, including new references to support information included due to reviewers’ comments. Reference number 71 is not included on the reference types but is the only one available of the oil spills situation on the study area, therefore, we request its inclusion since it was requested by one of the reviewers.**

**To Reviewers:**

**Reviewer #1**

**All grammatical suggestions were incorporated, thank you very much for the detail corrections and English grammar explanations; they were very useful.**

***INTRODUCTION***

**Line 70, 71-** Important FOR other shallow corals? that does not make sense. maybe the most important for shallow reef ecosystems? or most important OF the shallow reef corals? these topics mentioned need more discussion

**Yes, it was a grammatical mistake. It was also completed the paragraph**

**Line 88**- Is there information on what this means in terms of coral population parameters? It could be a helpful reference for discussing the results

**Yes, they have the same criteria used at IUCN red list**

**Line 91-** Please discuss why these parks are the most important. Even if it is only size, or management success.

**Coral reefs from both MPA, are the only true fully carbonate coastal reefs in the country whereby they are considered the most developed of the continental area of Venezuela. Extensive mangrove forests, seagrass meadows, and reef patches harbor high biodiversity in both MP´s**

**Line 94-** This Introduction is quite brief and many of the concepts could be further expanded upon both in terms of this study as well as previous literature. In the abstract you talked about spatial differences. This concept of why your looking at spatial differences could be introduced. Also, what are the different histories of the two MPA's you are sampling from, why were they chosen. Why are they the most important? How have the ecosystems responded differently to various disturbances. Also how are they different in terms of legality? As far as management practices, what types of management practices are put into place throughout the Caribbean for protecting Orbicella in particular? Do they exist?. The last point may be extra, but in any case, the ideas should to be discussed and explained more with outside literature.

**The suggested inclusion of paragraphs from the study site section to the introduction was done as well as the inclusion of information to improve the introduction.**

**Orbicella corals are under protection through the regulations mentioned (CITES SPAW Protocol, NMFS, ESA). In Venezuela there is not a direct protection of Orbicella species, but Marine Protected Areas have Management Plans, the Management Plan and Use Regulation (PORU by its acronym in Spanish) intend to protect the different ecosystems including coral reefs, so they are included when protecting the reefs. There are numerous laws and programs assigned to the protection of biodiversity in the country (Venezuelan Constitution, National Strategy of Biological Diversity, and the Law on Management of Biological Diversity among others) and they include coral reefs but not Orbicella directly.**

***STUDY SITE***

**Line 100-** This is where you should explain which sites were selected, how many from each zone etc etc

**Yes, this was included**

**Lines 102-117-** This is valuable background information that I suggest moving to the Introduction (see last comment in that section)

**Yes, agree, we moved to the introduction**

**Line 118-** This paragraph is no longer about the study area. I suggest to either start a new subsection under "data collection" or rename this subsection section to encompass the methodology

**Agree. The subtitle was changed to include both: study area and data collection**

**Line 121-**What part of the protocol did you modify?

**We did a continue record of substratum cover under the transect line, instead of record at 10 cm intervals points under the line, as the current protocol.**

**Line 123**-Usually coral surveys for AGRRA transects are 10 m long by 1 meter wide. not 10 m squared, is this the modification?

**It was a mistake, we corrected it**

**Line 123**-How were the variables mentioned above measured within each transect? How many transects were completed/collected at each site? what is the replicate number? I think slightly more detail in this process could be helpful but you can also write (see Lang et al., 2012 for further details).

**Considering that the complete method details are pointed out and explained at AGRRA PROTOCOL I included the suggestion of see Lang et al., 2012 for further details, but pertinent information was included on the Figure 1 (map) Legend**

**Line 124** Why?-Also, I would be careful with the wording here. These are repitions of data collection at different times. The temporal assessment comes with the data analysis portion. You can say "for the temporal assessment...". Also, why these months? Seasonally all 3 months are quite different.

**The months selected were related to the two seasons we have here in Venezuela (rain and drought) which have natural differences in temperature, winds, sediment discharges, etc. For this reason, we did the assessment in different months of the year.**

**Line 136**- Was this based on diseases?

**Concerning this question- if coral health status was based on diseases, the answer is yes, but is also related to the percentage of tissue mortality, so to avoid confusion I changed the phrase, focused on diseases.**

**Lines 136 and 138-** I suggest this should be in the new subsections.

**Agree, two new subsections were included in Data Analysis section (1) Live cover, mortality and diseases and (2) Colonies size structure). We decided to include two instead of three because some subjects were related to each other.**

**Line 140**- and faveolata?

**In case of *O. faveolata* the size of the colonies was plotted considering the reproductive size reported: > 100 cm maximum diameter**

**Line 146-**what test was performed?

**A Permutational analysis of variance (PERMANOVA) for density of reproductive and non-reproductive colonies were performed between sectors**

***RESULTS***

**Reviewer #1**

**All grammatical suggestions were incorporated, including the request of clarify sentences.**

**Line 208** Finally, regarding colony size and mortality, we found a positive relationship for O. faveolata (was this significant?)

**A new analysis was included to have a better understanding of the question about the relation between size and mortality**

**Reviewer #3**

**A new analysis was included to have a better understanding of the question about the relation between size and mortality**

***DISCUSSION***

**Line 221:** Was less affected by the factors relating to the massive die-off? had reduced mortality during the event?

The massive die-off was the consequence of causal factors, not the factor causing the event itself**.**

**Yes, the massive die-off was the consequence. Coral reefs from Sombrero had a** **lower mortality comparing to the other coral reefs from Morrocoy and Cayo Norte was not affected**

I think this deserves to be a sentence. Most likely, the whole thing could be 2-3 sentences. That way you can explain more clearly.

**Yes, it was included**

Is there a theory on why it was unaffected? Or was that due to currents too? I suggest rewriting for clarity.

**In case of Cuare, no direct causes have been identified, but the direction and intensity of the currents could be related**

**Line 235-** taking which? also specify the years of you are referencing a decrease. Was this since 1996? Or was this from the results here?

**Taking the studies mentioned in paragraph above as reference, reefs of MNP had a decrease in *O. annularis* live cover in most reefs since years 1999, 2000, 2004 and 2005 to years of the current study (2018-2020).**

**Line 242** "Key" may be better for a broader audience as it is more widely used whereas cay is a regionally specific term for only some parts of the Caribbean.

**Ok, it was included**

**Line 250** I recommend restructuring this sentence for clarity.

It was restructured

**Line 260** tend (or tended) to have -the tense depends on whether you are discussing the results of your study or general literature. Present if discussing literature (which I believe you are doing here)

**Yes, it will be tense**

**Line 299**. but were diseases only looked at on the Orbicella genus? Or did you look at all diseases found within a transect?

Only the Orbicella colonies were included

**Line 315** I'm not sure if this fits within the sentence. Either discuss the issues separately or keep the description of the invasive species brief. Also climate change projections are not the cause of the temperature increasing. this last part needs to be revised

**Agree, they were changed**

**Line 318** there needs to be a concise sentence regarding how the goal of the study was met, and what the overall results of the status of Orbicella are in these areas.

**Line 324** I am still not clear why especially this sector?

**As it was mentioned, the center sector is the area in Morrocoy that harbors the *Orbicella* colonies in better conditions,**

**Line 335** For a lot of these journals there are abbreviations that should be used (look at guidelines).

**Yes, they were all included**

***FIGURES***

**Line 4** It is difficult to make out the 3 different refuge sectors in the bottom. At first it looks like no sites are within the Cuare WR

**Agree, this was improved on the new map**

**Line 110.** color key, third from the top Unknow -> Unknown

**We didn´t understood this comment**

**Reviewer #2**

Conclusions must be based on the results obtained. Of course, it is important to make these observations, but I would suggest shortening this paragraph a bit.

**Agree, the paragraph was adjusted**

**Reviewer #3**

**English language and Submission Guidelines**

The excessive use of the word “for” in the data analysis sections should be reduced. It is important to standardize terms such as “cover,” “live coral cover,” and “coral cover” throughout the manuscript.

After mentioning the full names of the species Orbicella faveolata and Orbicella annularis, the abbreviations “O. faveolata” and “O. annularis” should be used in the rest of the document.

A review of the writing and punctuation throughout the document is necessary. The pages are not numbered, and the text is not double-spaced. Additionally, any spacing issues in the text should be corrected.

The citations and references do not follow the "Vancouver" style used by PLOS ONE. Furthermore, the figure legends are not inserted correctly after the first paragraph where each figure is cited

**Agree, they were all corrected and adjusted to the journal Guidelines**

Lines 137, 146, 176, 177, 180, 184

**All the changes suggested were included**

***MATERIALS AND METHODS***

It is crucial to clarify in the methodology which parameters defined the "status of Orbicella." For instance, in line 118, it states, “We assessed the following variables: density, cover, size structure, and mortality.” Improving the wording in this section would be beneficial.

**Yes, the status of *Orbicella* was assessed considering the following variables: density, live coral cover, size structure, and mortality.**

**In lines 120 and 121, it mentions,** “We use a modified version of the AGRRA Protocol 5.5.” It is necessary to specify what this modification entails.

**We did a continue record of substratum cover under the transect line, instead of record at 10 cm intervals points under the line, as the current protocol.**

**In lines 132 to 136**, it is important to provide a more detailed description of the analyses, specifying what each one (ANOVA and PERMANOVA) was used for and for which species specifically

*The materials and methods section were arranged in subsections to clarify*

***Agree, it was included in the main text***

***DATA ANALYSIS***

*It is important to provide sample sizes or the number of colonies analyzed for each year and in total. Furthermore, the databases used for the analyses should be attached as supplementary information or deposited in an appropriate public repository.*

**Agree, they were included and the database was sent as a Supporting Information file**

*The selected statistical methods are generally appropriate, especially PERMANOVA for spatial and temporal comparisons. However, since ecological data often do not meet the assumptions of normality and homogeneity of variance, it is recommended to verify that the data fulfill these assumptions for ANOVA.*

**As mentioned in the text:**

**Permutational analysis of variance (PERMANOVA) was used to analyzed: i) live cover of both Orbicella species, ii) tissue mortality (recent,	old,and total) of O.faveolata colonies and iii) density of reproductive and non-reproductive sizes of O. faveolata. The PERMANOVA mixed-effect linear model was done using two fixed factors (sampling period and sector) and a random factor (reef) nested within sector based on Euclidian distance. Analysis of lineal regression was used to estimate the relationship between total mortality (%) and sizes of Orbicella faveolata colonies at each period evaluates; and an analysis of variance was used to assess the lineal regression model fits,** **after testing assumptions using Shapiro-Wilk normality test, Breusch-Pagan test and Durbin-Watson test**

*Including additional multivariate analyses, such as PCoA or CCA, could provide a deeper understanding of the status of Orbicella. If possible, conducting time series analyses could assess trends in coral health parameters, including seasonal effects.*

**We decided to not include additional multivariate analyses because for an analysis such as PCoA or CCA, we would need a greater number of environmental variables or consider the values of the complete benthic analysis (benthic coverage).** **In addition, to be able to do a time series analysis, we would need the same transects in the four samples to follow up and evaluate trends, so we consider it is more appropriate to evaluate the seasonal effect considering the four samples within a fixed factor in the PERMANOVA analysis.**

***RESULTS***

**Line 186:** it is necessary to clarify what is meant by “prevalence

**Yes, this was included in data analysis section, subsection *Live coral cover, mortality and diseases***

**Line 155,** the percentage of “live coral cover” for O. faveolata should be mentioned.

**It was included**

**Line 196,** *“lower frequency” needs to specify what it is referring to.*

**Lines 155 to 157:** it is important to include key values from the PERMANOVA analysis, such as the F-value, p-value, and the percentage of variation explained for each relevant factor.

**It was included**

**Line 166**, change (Fig. 3) and move it to the end of the sentence in line 169.

***It was changed***

***DISCUSSION***

*In lines 219 and 220, it states, “The reefs where Orbicella colonies are in better health conditions nowadays are the same healthier more than 20 years ago.” The ecological and conservation implications of this statement should be discussed.*

***Agree, it was included***

***Line 311****,* mention the year of the recurrent oil spills and add the corresponding citation.

***Yes, they were included (References number 71 and 72). For year 2024, the reference is a technical report that doesn’t fit the reference types included on the journal guidelines, but is the only one available of the oil spills situation on the study area for 2024, therefore, we request its inclusion since it was requested by one of the reviewers.***

***Supplementary Tables***

*The results from Supplementary Table 4 support the claim that the density of reproductive colonies varies among sectors. Clarity could be improved by including in the text the proportion of explained* *variability (%CV), the F-value, and the p-value.*

**Agree, they were included**

*The information needs to be organized appropriately. For example, lines 128 and 129 in the data analysis section should be moved to the introduction as part of the study objectives. The initial paragraph in the data analysis section should also be restructured*

**Agree, changes were made to improve the manuscript**

***In lines 242 and 243****, translate “(Norte, Sur and Medio)” into English*

It was changed but in other cases it was keep as norte and sur, because they are proper names

*.*
